# 9-Ethynyl-9*H*-carbazole

**DOI:** 10.1107/S1600536812007143

**Published:** 2012-02-24

**Authors:** Hideyuki Tabata, Tsunehisa Okuno

**Affiliations:** a Department of Material Science and Chemistry, Wakayama University, Sakaedani, Wakayama, 640-8510, Japan

## Abstract

The title compound, C_14_H_9_N, is the second crystallographically characterized example of an ynamine with an H atom in the C-terminal position. There are two independent mol­ecules (*A* and *B*) in the asymmetric unit. The structures of both mol­ecules are essentially planar (r.m.s. deviation = 0.0312 and 0.0152 Å). The N—C_*sp*_ bond lengths are 1.353 (4) and 1.350 (4) Å, and those of the acetyl­ene bonds are 1.189 (4) and 1.190 (4) Å. The C_*sp*_—H bond lengths are 0.95 (5) and 0.97 (4) Å. These geometries are consistent with those of the previously reported ynamine characterized by crystallography. In the crystal, the mol­ecules stack along the *c* axis, forming two kinds of columnar structures. The acetyl­ene C atoms of mol­ecule *A* have a short contact [3.341 (4) Å and 3.396 (4) Å] with an adjacent mol­ecule *A* at the C—C bond of the fused part, which originates in π–π stacking inter­action; no remarkable spatial contact is recognized within the stacking of mol­ecule *B*.

## Related literature

For the preparation of the title compound, see: Cuniberti *et al.* (1996 ▶). For the related structure of a diacetyl­ene compound having 9-carbazolyl groups at both ends, see: Mayerle & Flandera (1978 ▶). For the related structure of an ynamine compound that carrys an H atom at an acetyl­ene terminal, see: Tabata & Okuno (2011 ▶). For related structures of ynamine compounds, see: Galli *et al.* (1988 ▶, 1989 ▶); Okuno *et al.* (2006 ▶); Tabata *et al.* (2012 ▶).
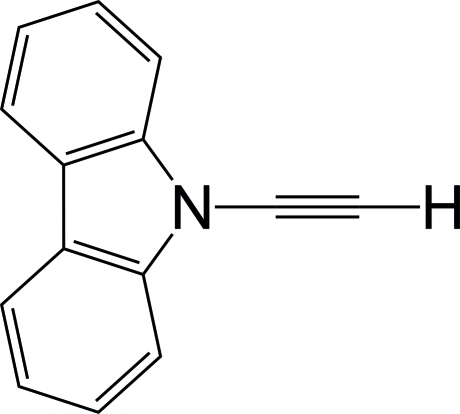


## Experimental

### 

#### Crystal data

C_14_H_9_N
*M* *_r_* = 191.23
Orthorhombic, 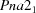

*a* = 23.642 (5) Å
*b* = 16.171 (4) Å
*c* = 5.1523 (11) Å
*V* = 1969.8 (8) Å^3^
*Z* = 8
Mo *K*α radiation
μ = 0.08 mm^−1^
*T* = 93 K
0.15 × 0.07 × 0.04 mm

#### Data collection

Rigaku Saturn724+ diffractometer
Absorption correction: numerical (*NUMABS*; Rigaku, 1999 ▶) *T*_min_ = 0.994, *T*_max_ = 0.997
16048 measured reflections
2515 independent reflections
2257 reflections with *F* ^2^ > 2σ(*F* ^2^)
*R*_int_ = 0.068

#### Refinement

*R*[*F* ^2^ > 2σ(*F* ^2^)] = 0.050
*wR*(*F* ^2^) = 0.134
*S* = 1.10
2513 reflections
279 parameters
1 restraint
H atoms treated by a mixture of independent and constrained refinement
Δρ_max_ = 0.19 e Å^−3^
Δρ_min_ = −0.23 e Å^−3^

### 

Data collection: *CrystalClear* (Rigaku, 2008 ▶); cell refinement: *CrystalClear*; data reduction: *CrystalClear*; program(s) used to solve structure: *SIR92* (Altomare, *et al.*, 1994 ▶); program(s) used to refine structure: *SHELXL97* (Sheldrick, 2008 ▶); molecular graphics: *ORTEP-3* (Farrugia, 1997 ▶); software used to prepare material for publication: *CrystalStructure* (Rigaku, 2010 ▶).

## Supplementary Material

Crystal structure: contains datablock(s) global, I. DOI: 10.1107/S1600536812007143/nk2144sup1.cif

Structure factors: contains datablock(s) I. DOI: 10.1107/S1600536812007143/nk2144Isup2.hkl

Supplementary material file. DOI: 10.1107/S1600536812007143/nk2144Isup3.cml

Additional supplementary materials:  crystallographic information; 3D view; checkCIF report

## Figures and Tables

**Fig. 1.**
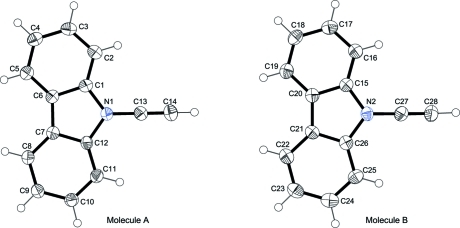
The asymmetric unit of the title compound with displacement ellipsoids drawn at the 50% probability level.

**Fig. 2.**
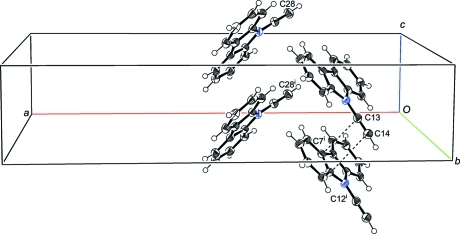
A view of the molecular stacking structure. [Symmetry codes: (i) x, y, z - 1.].

## supplementary crystallographic information

### Comment

Ynamines, where amino groups connect to acetylene groups, are known to be unstable because of their high reactivity. Therefore, the reports of the crystal structures were limited to rather stable ynamines (Galli *et al.*, 1988; Galli *et al.*, 1989; Mayerle & Flandera, 1978; Okuno *et al.*, 2006; Tabata *et al.*, 2012) which carry some substituents except H atom on all C– and N-terminals. Recently, we reported the crystal structure of *N*^1^,*N*^4^-diethynyl -*N*^1^,*N*^4^-diphenylbenzene-1,4-diamine, which is the first crystallographic report of an ynamine with a C-terminal H atom (Tabata & Okuno, 2011). We report herein the crystal structure of 9-ethynyl-9*H*-carbazole which is the second example of ynamines with the C-terminal H atom.

There are two independent molecules (Molecule A and B) in the unit cell (Fig. 1). The structures of both molecules are planar (N1/C1—C14 plane: r.m.s. deviation = 0.0312 Å, N2/C15—C28 plane: r.m.s. deviation = 0.0152 Å).

The bond lengths of N1—C13 and N2—C27 are 1.353 (4) Å and 1.350 (4) Å, respectively. Those of acetylene bonds in C13—C14 and C27—C28 are 1.189 (4) Å and 1.190 (4) Å. The C*_sp_*—H lengths of C14—H14 and C28—H28 are 0.95 (5) Å and 0.97 (4) Å, respectively. Although delocalization of lone pair of the N atoms is thought to shrink these bonds, the molecular geometries are consistent with those of the reported ynamines (Table 1).

The molecules stack along the *c* axis, forming two kinds of columnar structures. The spatial contact between Molecules A is recognized within the columnar stack. The acetylenic carbons of C13 and C14 show the short contact with C7^i^ [Symmetry codes:(i) *x*, *y*, *z* - 1.] and C12^i^, where the distances of C13···C7^i^ and C14···C12^i^ are 3.341 (4) Å and 3.396 (4) Å, respectively, indicating π-π stacking interaction. While remarkable spatial contact is not recognized within the stacking of Molecule B.

### Experimental

The title compound was prepared according to a published procedure (Cuniberti *et al.*, 1996). The purification of the compound was performed by gel permeation chromatography (GPC). The single crystals with sufficient quality for X-ray analysis were obtained by concentration of an ethereal solution in a refrigerator.

### Refinement

Friedel pairs were merged because the molecule itself was achiral and because there were not any anomalous scattering effects. The C-bound H atoms except two C*_sp_*—H atoms were placed at ideal positions and were refined as riding on their parent C atoms. *U*_iso_(H) values of the H atoms were set at 1.2*U*_eq_(parent atom). The C*_sp_*-bound H atoms were obtained from a difference Fourier map and were refined isotropically without any restrictions.

### Crystal data

**Table:**

C_14_H_9_N	*F*(000) = 800.00
*M**_r_* = 191.23	*D*_x_ = 1.290 Mg m^−^^3^
Orthorhombic, *P**n**a*2_1_	Mo *K*α radiation, λ = 0.71075 Å
Hall symbol: P 2c -2n	Cell parameters from 6052 reflections
*a* = 23.642 (5) Å	θ = 1.5–31.3°
*b* = 16.171 (4) Å	µ = 0.08 mm^−^^1^
*c* = 5.1523 (11) Å	*T* = 93 K
*V* = 1969.8 (8) Å^3^	Prism, colourless
*Z* = 8	0.15 × 0.07 × 0.04 mm

### Data collection

**Table:**

Rigaku Saturn724+ diffractometer	2257 reflections with *F*^2^ > 2σ(*F*^2^)
Detector resolution: 7.111 pixels mm^-1^	*R*_int_ = 0.068
ω scans	θ_max_ = 27.5°
Absorption correction: numerical (*NUMABS*; Rigaku, 1999)	*h* = −25→30
*T*_min_ = 0.994, *T*_max_ = 0.997	*k* = −20→21
16048 measured reflections	*l* = −6→6
2515 independent reflections	

### Refinement

**Table:**

Refinement on *F*^2^	Secondary atom site location: difference Fourier map
*R*[*F*^2^ > 2σ(*F*^2^)] = 0.050	Hydrogen site location: inferred from neighbouring sites
*wR*(*F*^2^) = 0.134	H atoms treated by a mixture of independent and constrained refinement
*S* = 1.10	*w* = 1/[σ^2^(*F*_o_^2^) + (0.0842*P*)^2^ + 0.1152*P*] where *P* = (*F*_o_^2^ + 2*F*_c_^2^)/3
2513 reflections	(Δ/σ)_max_ < 0.001
279 parameters	Δρ_max_ = 0.19 e Å^−^^3^
1 restraint	Δρ_min_ = −0.23 e Å^−^^3^
Primary atom site location: structure-invariant direct methods	

### Special details

**Table:**

Refinement. Refinement was performed using all reflections except for 2 with very negative *F*^2^. The weighted *R*-factor (*wR*) and goodness of fit (*S*) are based on *F*^2^. *R*-factor (gt) are based on *F*. The threshold expression of *F*^2^ > 2.0 σ(*F*^2^) is used only for calculating *R*-factor (gt).

### Fractional atomic coordinates and isotropic or equivalent isotropic displacement parameters (Å^2^)

**Table:**

	*x*	*y*	*z*	*U*_iso_*/*U*_eq_	
N1	0.17927 (8)	0.32008 (12)	0.3093 (5)	0.0276 (5)	
N2	0.39976 (8)	0.18688 (12)	1.0812 (5)	0.0284 (5)	
C1	0.16682 (10)	0.23947 (14)	0.3946 (6)	0.0274 (6)	
C2	0.12609 (10)	0.18553 (15)	0.3004 (6)	0.0302 (6)	
C3	0.12131 (10)	0.10968 (15)	0.4266 (6)	0.0326 (6)	
C4	0.15618 (11)	0.08922 (15)	0.6371 (6)	0.0335 (6)	
C5	0.19701 (11)	0.14394 (14)	0.7252 (6)	0.0293 (6)	
C6	0.20259 (9)	0.22029 (14)	0.6041 (6)	0.0262 (5)	
C7	0.23825 (10)	0.29183 (14)	0.6489 (5)	0.0263 (5)	
C8	0.27939 (10)	0.31096 (15)	0.8342 (6)	0.0288 (6)	
C9	0.30345 (11)	0.38944 (15)	0.8297 (6)	0.0310 (6)	
C10	0.28710 (10)	0.44797 (15)	0.6461 (6)	0.0309 (6)	
C11	0.24655 (10)	0.43051 (15)	0.4599 (6)	0.0306 (6)	
C12	0.22252 (9)	0.35269 (14)	0.4671 (6)	0.0260 (5)	
C13	0.15250 (10)	0.36112 (15)	0.1163 (6)	0.0294 (6)	
C14	0.12871 (11)	0.39684 (18)	−0.0533 (7)	0.0363 (6)	
C15	0.43363 (10)	0.15633 (15)	0.8781 (6)	0.0271 (6)	
C16	0.43046 (10)	0.08119 (16)	0.7514 (6)	0.0325 (6)	
C17	0.46952 (11)	0.06664 (17)	0.5561 (6)	0.0348 (6)	
C18	0.51012 (10)	0.12635 (17)	0.4875 (6)	0.0341 (6)	
C19	0.51259 (10)	0.20088 (16)	0.6155 (6)	0.0327 (6)	
C20	0.47392 (9)	0.21683 (15)	0.8143 (6)	0.0279 (6)	
C21	0.46426 (10)	0.28721 (14)	0.9841 (6)	0.0283 (6)	
C22	0.48979 (11)	0.36437 (16)	1.0080 (7)	0.0335 (6)	
C23	0.46980 (11)	0.41830 (16)	1.1975 (7)	0.0371 (7)	
C24	0.42500 (11)	0.39549 (16)	1.3606 (6)	0.0363 (7)	
C25	0.39850 (10)	0.31955 (15)	1.3376 (6)	0.0322 (6)	
C26	0.41907 (10)	0.26691 (14)	1.1465 (6)	0.0281 (6)	
C27	0.35575 (11)	0.14677 (16)	1.1907 (6)	0.0329 (6)	
C28	0.31651 (11)	0.11167 (17)	1.2844 (7)	0.0391 (7)	
H2	0.1027	0.1996	0.1572	0.0363*	
H3	0.0938	0.0710	0.3687	0.0391*	
H4	0.1518	0.0372	0.7203	0.0402*	
H5	0.2209	0.1295	0.8664	0.0352*	
H8	0.2906	0.2713	0.9601	0.0345*	
H9	0.3315	0.4034	0.9542	0.0372*	
H10	0.3042	0.5011	0.6486	0.0370*	
H11	0.2358	0.4702	0.3332	0.0367*	
H14	0.1113 (15)	0.424 (3)	−0.197 (11)	0.070 (12)*	
H16	0.4027	0.0412	0.7963	0.0389*	
H17	0.4688	0.0153	0.4666	0.0418*	
H18	0.5361	0.1151	0.3513	0.0409*	
H19	0.5402	0.2409	0.5694	0.0392*	
H22	0.5202	0.3799	0.8975	0.0402*	
H23	0.4867	0.4712	1.2166	0.0445*	
H24	0.4125	0.4331	1.4901	0.0436*	
H25	0.3679	0.3042	1.4468	0.0386*	
H28	0.2863 (16)	0.080 (3)	1.364 (9)	0.077 (13)*	

### Atomic displacement parameters (Å^2^)

**Table:**

	*U*^11^	*U*^22^	*U*^33^	*U*^12^	*U*^13^	*U*^23^
N1	0.0266 (10)	0.0281 (10)	0.0280 (11)	0.0002 (8)	−0.0024 (9)	0.0021 (10)
N2	0.0235 (10)	0.0294 (10)	0.0322 (12)	−0.0012 (8)	−0.0005 (9)	0.0021 (10)
C1	0.0261 (11)	0.0256 (11)	0.0303 (14)	0.0010 (9)	0.0048 (10)	−0.0013 (11)
C2	0.0258 (12)	0.0343 (13)	0.0305 (13)	0.0019 (9)	0.0015 (11)	−0.0056 (12)
C3	0.0278 (12)	0.0300 (13)	0.0399 (16)	−0.0029 (10)	0.0029 (12)	−0.0049 (12)
C4	0.0323 (13)	0.0285 (12)	0.0397 (16)	−0.0002 (9)	0.0065 (12)	0.0007 (13)
C5	0.0308 (13)	0.0273 (11)	0.0299 (14)	0.0036 (10)	0.0033 (10)	−0.0007 (11)
C6	0.0234 (11)	0.0264 (11)	0.0288 (13)	0.0032 (9)	0.0052 (10)	0.0007 (11)
C7	0.0262 (11)	0.0283 (12)	0.0245 (13)	0.0013 (9)	0.0034 (10)	−0.0003 (10)
C8	0.0303 (12)	0.0297 (12)	0.0263 (12)	0.0034 (9)	−0.0005 (11)	0.0009 (11)
C9	0.0308 (12)	0.0332 (13)	0.0291 (13)	0.0015 (10)	−0.0030 (11)	−0.0007 (12)
C10	0.0317 (13)	0.0292 (12)	0.0316 (14)	−0.0018 (10)	−0.0000 (11)	−0.0002 (12)
C11	0.0314 (12)	0.0301 (12)	0.0301 (13)	−0.0009 (10)	0.0000 (11)	0.0042 (11)
C12	0.0246 (11)	0.0283 (12)	0.0250 (12)	0.0028 (9)	−0.0000 (10)	0.0009 (10)
C13	0.0268 (12)	0.0315 (12)	0.0298 (14)	0.0026 (10)	0.0005 (11)	−0.0024 (12)
C14	0.0346 (14)	0.0420 (15)	0.0322 (15)	0.0066 (12)	−0.0016 (13)	−0.0009 (13)
C15	0.0250 (12)	0.0297 (12)	0.0266 (13)	0.0039 (9)	−0.0024 (10)	0.0023 (11)
C16	0.0299 (13)	0.0310 (13)	0.0364 (15)	0.0010 (10)	−0.0026 (11)	0.0016 (12)
C17	0.0343 (13)	0.0363 (13)	0.0339 (16)	0.0060 (11)	−0.0044 (11)	−0.0037 (13)
C18	0.0285 (12)	0.0435 (14)	0.0302 (14)	0.0066 (11)	0.0008 (11)	0.0009 (13)
C19	0.0287 (13)	0.0351 (13)	0.0343 (14)	0.0021 (10)	0.0003 (12)	0.0049 (13)
C20	0.0248 (11)	0.0295 (12)	0.0295 (13)	0.0032 (9)	−0.0040 (11)	0.0040 (12)
C21	0.0282 (12)	0.0254 (11)	0.0313 (13)	0.0024 (9)	−0.0050 (11)	0.0024 (11)
C22	0.0304 (13)	0.0294 (12)	0.0407 (16)	0.0004 (10)	−0.0048 (12)	0.0035 (12)
C23	0.0363 (14)	0.0312 (13)	0.0437 (17)	0.0008 (10)	−0.0115 (13)	−0.0011 (13)
C24	0.0382 (14)	0.0348 (13)	0.0359 (15)	0.0095 (11)	−0.0111 (12)	−0.0023 (13)
C25	0.0301 (13)	0.0352 (14)	0.0311 (14)	0.0075 (10)	−0.0012 (11)	0.0005 (13)
C26	0.0256 (12)	0.0290 (12)	0.0298 (14)	0.0026 (9)	−0.0051 (10)	0.0035 (12)
C27	0.0299 (13)	0.0361 (13)	0.0326 (15)	0.0014 (10)	0.0017 (11)	0.0010 (12)
C28	0.0361 (15)	0.0412 (15)	0.0398 (16)	−0.0029 (12)	0.0056 (13)	0.0040 (14)

### Geometric parameters (Å, º)

**Table:**

N1—C1	1.407 (3)	C20—C21	1.454 (4)
N1—C12	1.409 (4)	C21—C22	1.392 (4)
N1—C13	1.353 (4)	C21—C26	1.396 (4)
N2—C15	1.407 (4)	C22—C23	1.392 (5)
N2—C26	1.413 (3)	C23—C24	1.401 (4)
N2—C27	1.350 (4)	C24—C25	1.384 (4)
C1—C2	1.387 (4)	C25—C26	1.390 (4)
C1—C6	1.406 (4)	C27—C28	1.190 (4)
C2—C3	1.393 (4)	C2—H2	0.950
C3—C4	1.402 (4)	C3—H3	0.950
C4—C5	1.386 (4)	C4—H4	0.950
C5—C6	1.390 (4)	C5—H5	0.950
C6—C7	1.450 (4)	C8—H8	0.950
C7—C8	1.398 (4)	C9—H9	0.950
C7—C12	1.409 (4)	C10—H10	0.950
C8—C9	1.391 (4)	C11—H11	0.950
C9—C10	1.393 (4)	C14—H14	0.95 (5)
C10—C11	1.385 (4)	C16—H16	0.950
C11—C12	1.381 (4)	C17—H17	0.950
C13—C14	1.189 (4)	C18—H18	0.950
C15—C16	1.381 (4)	C19—H19	0.950
C15—C20	1.405 (4)	C22—H22	0.950
C16—C17	1.386 (4)	C23—H23	0.950
C17—C18	1.407 (4)	C24—H24	0.950
C18—C19	1.375 (4)	C25—H25	0.950
C19—C20	1.397 (4)	C28—H28	0.97 (4)
			
N1···C5	3.589 (4)	C24···H22^ii^	3.5747
N1···C8	3.597 (4)	C24···H23^xiv^	3.5162
N2···C19	3.595 (4)	C25···H9	2.8718
N2···C22	3.593 (4)	C25···H8	3.3007
C1···C4	2.744 (4)	C26···H9	3.1834
C1···C14	3.552 (4)	C26···H8	3.1854
C2···C5	2.838 (4)	C27···H8	2.7997
C2···C13	3.058 (4)	C28···H5	3.1358
C3···C6	2.780 (4)	C28···H8	3.1356
C5···C8	3.377 (4)	C28···H10^vii^	3.4384
C6···C13	3.593 (4)	C28···H11^vii^	3.4880
C7···C10	2.777 (4)	C28···H14^xv^	3.49 (4)
C7···C13	3.591 (4)	C28···H16^ii^	3.5222
C8···C11	2.839 (4)	H9···C3^viii^	3.5205
C9···C12	2.740 (4)	H9···C4^viii^	3.1631
C11···C13	3.056 (4)	H9···C11^ii^	3.3195
C12···C14	3.552 (4)	H9···C23	3.5093
C15···C18	2.749 (4)	H9···C24	3.0469
C15···C28	3.545 (4)	H9···C25	2.8718
C16···C19	2.830 (4)	H9···C26	3.1834
C16···C27	3.060 (4)	H9···H3^viii^	3.2638
C17···C20	2.771 (4)	H9···H4^viii^	2.5924
C19···C22	3.372 (4)	H9···H11^ii^	3.1793
C20···C27	3.585 (4)	H9···H24^i^	3.1003
C21···C24	2.773 (4)	H9···H24	3.3943
C21···C27	3.588 (4)	H9···H25^i^	3.1847
C22···C25	2.840 (4)	H9···H25	3.1227
C23···C26	2.739 (4)	H2···C4^i^	3.4596
C25···C27	3.066 (4)	H2···C5^i^	3.2771
C26···C28	3.562 (4)	H2···C19^v^	3.5638
N1···C8^i^	3.409 (4)	H2···C21^iii^	3.3983
N2···C17^ii^	3.534 (4)	H2···C22^iii^	2.9636
N2···C18^ii^	3.485 (4)	H2···H5^i^	3.3673
C2···C5^i^	3.471 (4)	H2···H18^v^	3.5299
C5···C2^ii^	3.471 (4)	H2···H19^iii^	3.5041
C7···C13^ii^	3.341 (4)	H2···H19^v^	2.7597
C7···C14^ii^	3.456 (4)	H2···H22^iii^	2.6920
C8···N1^ii^	3.409 (4)	H3···C22^iii^	3.2555
C8···C12^ii^	3.591 (4)	H3···C23^vi^	3.3511
C8···C13^ii^	3.431 (4)	H3···C23^iii^	3.0672
C9···C11^ii^	3.576 (4)	H3···H9^vi^	3.2638
C9···C25^i^	3.571 (4)	H3···H10^vi^	2.8942
C11···C9^i^	3.576 (4)	H3···H19^v^	3.4537
C12···C8^i^	3.591 (4)	H3···H22^iii^	3.0919
C12···C14^ii^	3.396 (4)	H3···H22^v^	3.3300
C13···C7^i^	3.341 (4)	H3···H23^vi^	3.0730
C13···C8^i^	3.431 (4)	H3···H23^iii^	2.7371
C14···C7^i^	3.456 (4)	H3···H24^xvi^	2.9664
C14···C12^i^	3.396 (4)	H4···C9^vi^	3.2986
C14···C19^iii^	3.598 (4)	H4···C10^vi^	3.5951
C16···C27^i^	3.549 (4)	H4···C10^vii^	2.9973
C17···N2^i^	3.534 (4)	H4···C11^vii^	3.2062
C17···C27^i^	3.530 (4)	H4···C23^vi^	3.4598
C18···N2^i^	3.485 (4)	H4···C24^vi^	3.0112
C18···C26^i^	3.590 (4)	H4···H9^vi^	2.5924
C19···C14^iv^	3.598 (4)	H4···H10^vi^	3.1783
C19···C26^i^	3.445 (4)	H4···H10^vii^	2.5092
C20···C25^i^	3.460 (4)	H4···H11^vii^	2.9296
C25···C9^ii^	3.571 (4)	H4···H22^v^	3.5088
C25···C20^ii^	3.460 (4)	H4···H23^vi^	3.4438
C26···C18^ii^	3.590 (4)	H4···H24^vi^	2.6598
C26···C19^ii^	3.445 (4)	H5···C1^ii^	3.4934
C27···C16^ii^	3.549 (4)	H5···C2^ii^	3.2927
C27···C17^ii^	3.530 (4)	H5···C10^vii^	3.2748
N1···H2	2.7731	H5···C11^vii^	3.3429
N1···H11	2.7734	H5···C28	3.1358
N1···H14	3.49 (5)	H5···H2^ii^	3.3673
N2···H28	3.51 (4)	H5···H10^vii^	2.6018
N2···H16	2.7766	H5···H11^vii^	2.7773
N2···H25	2.7780	H5···H28^i^	3.1172
C1···H3	3.2278	H5···H28	3.0974
C1···H5	3.2722	H8···N1^ii^	3.2851
C2···H4	3.2868	H8···N2	2.9849
C3···H5	3.2830	H8···C12^ii^	3.3388
C4···H2	3.3012	H8···C25	3.3007
C5···H3	3.2733	H8···C26	3.1854
C5···H8	3.2571	H8···C27	2.7997
C6···H2	3.3156	H8···C28	3.1356
C6···H4	3.2510	H8···H25^i^	3.2573
C6···H8	2.8946	H8···H25	3.1470
C7···H9	3.2545	H10···C2^viii^	3.4960
C7···H5	2.8846	H10···C3^viii^	2.8698
C7···H11	3.3118	H10···C4^ix^	3.1394
C8···H5	3.2493	H10···C4^viii^	3.0398
C8···H10	3.2734	H10···C5^ix^	3.1771
C9···H11	3.2879	H10···C28^ix^	3.4384
C10···H8	3.2838	H10···H3^viii^	2.8942
C11···H9	3.2735	H10···H4^ix^	2.5092
C12···H8	3.2829	H10···H4^viii^	3.1783
C12···H10	3.2193	H10···H5^ix^	2.6018
C13···H2	2.8733	H10···H28^ix^	2.7289
C13···H11	2.8698	H10···H24^i^	2.9051
C14···H2	3.4250	H11···C4^ix^	3.3544
C14···H11	3.4321	H11···C5^ix^	3.2757
C15···H17	3.2237	H11···C9^i^	3.3157
C15···H19	3.2787	H11···C28^ix^	3.4880
C16···H18	3.2843	H11···H9^i^	3.1793
C17···H19	3.2775	H11···H4^ix^	2.9296
C18···H16	3.2982	H11···H5^ix^	2.7773
C19···H17	3.2660	H11···H28^xvii^	3.0481
C19···H22	3.2439	H11···H28^ix^	3.3065
C20···H16	3.3032	H11···H16^ix^	3.4742
C20···H18	3.2492	H14···N1^i^	3.45 (5)
C20···H22	2.8864	H14···C12^i^	3.35 (4)
C21···H19	2.8895	H14···C16^ix^	3.58 (5)
C21···H23	3.2515	H14···C17^ix^	3.27 (4)
C21···H25	3.3093	H14···C17^iii^	3.59 (4)
C22···H19	3.2421	H14···C18^iii^	3.00 (5)
C22···H24	3.2777	H14···C19^iii^	3.23 (4)
C23···H25	3.2957	H14···C28^xvii^	3.49 (4)
C24···H22	3.2889	H14···H28^xvii^	3.52 (6)
C25···H23	3.2799	H14···H16^xvii^	3.2455
C26···H22	3.2703	H14···H16^ix^	3.1881
C26···H24	3.2220	H14···H17^ix^	2.5465
C27···H16	2.8767	H14···H18^iii^	2.9963
C27···H25	2.8819	H14···H18^v^	3.3959
C28···H16	3.4314	H14···H19^iii^	3.3732
C28···H25	3.4451	H28···C4^ii^	3.38 (4)
H9···H8	2.3442	H28···C5^ii^	2.99 (5)
H9···H10	2.3230	H28···C6^ii^	3.25 (4)
H2···H3	2.3572	H28···C10^vii^	2.98 (4)
H3···H4	2.3357	H28···C11^vii^	3.29 (5)
H4···H5	2.3369	H28···H5	3.0974
H5···H8	2.8668	H28···H5^ii^	3.1172
H10···H11	2.3466	H28···H10^vii^	2.7289
H16···H17	2.3467	H28···H11^vii^	3.3065
H17···H18	2.3421	H28···H11^xv^	3.0481
H18···H19	2.3270	H28···H14^xv^	3.52 (6)
H19···H22	2.8515	H28···H16^ii^	3.5968
H22···H23	2.3471	H16···C13^vii^	3.3231
H23···H24	2.3337	H16···C14^vii^	3.0406
H24···H25	2.3470	H16···C18^x^	3.5442
N1···H8^i^	3.2851	H16···C28^i^	3.5222
N1···H14^ii^	3.45 (5)	H16···H11^vii^	3.4742
N1···H18^v^	3.5501	H16···H14^vii^	3.1881
N2···H8	2.9849	H16···H14^xv^	3.2455
C1···H5^i^	3.4934	H16···H28^i^	3.5968
C1···H19^v^	3.1420	H16···H17^x^	3.2906
C2···H5^i^	3.2927	H16···H18^x^	2.9259
C2···H10^vi^	3.4960	H17···C14^vii^	2.9997
C2···H19^v^	2.7310	H17···C16^xi^	3.0544
C2···H22^iii^	3.4204	H17···C17^xi^	2.8900
C3···H9^vi^	3.5205	H17···C18^xi^	3.4039
C3···H10^vi^	2.8698	H17···C18^x^	3.5629
C3···H19^v^	3.1708	H17···H14^vii^	2.5465
C3···H22^v^	3.4111	H17···H16^xi^	3.2906
C4···H9^vi^	3.1631	H17···H17^xi^	3.0083
C4···H2^ii^	3.4596	H17···H17^x^	3.0083
C4···H10^vi^	3.0398	H17···H18^x^	2.8958
C4···H10^vii^	3.1394	H18···N1^xii^	3.5501
C4···H11^vii^	3.3544	H18···C13^xii^	3.0312
C4···H28^i^	3.38 (4)	H18···C14^xii^	3.0295
C4···H22^v^	3.5202	H18···C15^i^	3.5012
C4···H24^vi^	3.5100	H18···C16^xi^	3.3110
C5···H2^ii^	3.2771	H18···C17^xi^	3.3116
C5···H10^vii^	3.1771	H18···C20^i^	3.5393
C5···H11^vii^	3.2757	H18···H2^xii^	3.5299
C5···H28^i^	2.99 (5)	H18···H14^xii^	3.3959
C6···H28^i^	3.25 (4)	H18···H14^iv^	2.9963
C7···H25^i^	3.2424	H18···H16^xi^	2.9259
C8···H25^i^	2.8931	H18···H17^xi^	2.8958
C9···H4^viii^	3.2986	H19···C1^xii^	3.1420
C9···H11^ii^	3.3157	H19···C2^xii^	2.7310
C9···H24^i^	3.1951	H19···C3^xii^	3.1708
C9···H25^i^	2.8481	H19···C21^i^	3.5884
C10···H4^ix^	2.9973	H19···H2^xii^	2.7597
C10···H4^viii^	3.5951	H19···H2^iv^	3.5041
C10···H5^ix^	3.2748	H19···H3^xii^	3.4537
C10···H28^ix^	2.98 (4)	H19···H14^iv^	3.3732
C10···H24^i^	3.0813	H22···C2^iv^	3.4204
C10···H25^i^	3.1788	H22···C3^xii^	3.4111
C11···H9^i^	3.3195	H22···C4^xii^	3.5202
C11···H4^ix^	3.2062	H22···C23^xiii^	3.4307
C11···H5^ix^	3.3429	H22···C24^i^	3.5747
C11···H28^ix^	3.29 (5)	H22···H2^iv^	2.6920
C11···H25^i^	3.5216	H22···H3^xii^	3.3300
C12···H8^i^	3.3388	H22···H3^iv^	3.0919
C12···H14^ii^	3.35 (4)	H22···H4^xii^	3.5088
C12···H25^i^	3.5259	H22···H23^xiii^	2.5871
C13···H16^ix^	3.3231	H22···H24^i^	3.4094
C13···H18^v^	3.0312	H22···H24^xiii^	3.4508
C14···H16^ix^	3.0406	H23···C22^xiv^	3.1033
C14···H17^ix^	2.9997	H23···C23^xiii^	3.3762
C14···H18^v^	3.0295	H23···C23^xiv^	3.2230
C15···H18^ii^	3.5012	H23···C24^xiii^	3.5162
C16···H14^vii^	3.58 (5)	H23···H3^viii^	3.0730
C16···H17^x^	3.0544	H23···H3^iv^	2.7371
C16···H18^x^	3.3110	H23···H4^viii^	3.4438
C17···H14^vii^	3.27 (4)	H23···H22^xiv^	2.5871
C17···H14^iv^	3.59 (4)	H23···H23^xiii^	2.8099
C17···H17^x^	2.8900	H23···H23^xiv^	2.8099
C17···H18^x^	3.3116	H23···H24^xiii^	3.0706
C18···H14^iv^	3.00 (5)	H24···C4^viii^	3.5100
C18···H16^xi^	3.5442	H24···C9^ii^	3.1951
C18···H17^xi^	3.5629	H24···C10^ii^	3.0813
C18···H17^x^	3.4039	H24···C22^ii^	3.4196
C19···H2^xii^	3.5638	H24···H9	3.3943
C19···H14^iv^	3.23 (4)	H24···H9^ii^	3.1003
C20···H18^ii^	3.5393	H24···H3^xviii^	2.9664
C20···H25^i^	3.4451	H24···H4^viii^	2.6598
C21···H2^iv^	3.3983	H24···H10^ii^	2.9051
C21···H19^ii^	3.5884	H24···H22^ii^	3.4094
C21···H25^i^	3.5965	H24···H22^xiv^	3.4508
C22···H2^iv^	2.9636	H24···H23^xiv^	3.0706
C22···H3^iv^	3.2555	H25···C7^ii^	3.2424
C22···H23^xiii^	3.1033	H25···C8^ii^	2.8931
C22···H24^i^	3.4196	H25···C9^ii^	2.8481
C23···H9	3.5093	H25···C10^ii^	3.1788
C23···H3^viii^	3.3511	H25···C11^ii^	3.5216
C23···H3^iv^	3.0672	H25···C12^ii^	3.5259
C23···H4^viii^	3.4598	H25···C20^ii^	3.4451
C23···H22^xiv^	3.4307	H25···C21^ii^	3.5965
C23···H23^xiii^	3.2230	H25···H9	3.1227
C23···H23^xiv^	3.3762	H25···H9^ii^	3.1847
C24···H9	3.0469	H25···H8	3.1470
C24···H4^viii^	3.0112	H25···H8^ii^	3.2573
			
C1—N1—C12	108.6 (2)	C22—C23—C24	120.8 (3)
C1—N1—C13	125.9 (2)	C23—C24—C25	121.6 (3)
C12—N1—C13	125.5 (2)	C24—C25—C26	116.5 (3)
C15—N2—C26	108.3 (2)	N2—C26—C21	108.7 (3)
C15—N2—C27	125.5 (3)	N2—C26—C25	128.1 (3)
C26—N2—C27	126.1 (3)	C21—C26—C25	123.3 (3)
N1—C1—C2	128.2 (3)	N2—C27—C28	179.1 (3)
N1—C1—C6	108.6 (2)	C1—C2—H2	121.731
C2—C1—C6	123.2 (3)	C3—C2—H2	121.729
C1—C2—C3	116.5 (3)	C2—C3—H3	119.295
C2—C3—C4	121.4 (3)	C4—C3—H3	119.293
C3—C4—C5	120.8 (3)	C3—C4—H4	119.580
C4—C5—C6	119.1 (3)	C5—C4—H4	119.601
C1—C6—C5	118.9 (3)	C4—C5—H5	120.456
C1—C6—C7	107.2 (2)	C6—C5—H5	120.447
C5—C6—C7	133.8 (3)	C7—C8—H8	120.803
C6—C7—C8	133.6 (3)	C9—C8—H8	120.815
C6—C7—C12	107.4 (2)	C8—C9—H9	119.414
C8—C7—C12	118.9 (3)	C10—C9—H9	119.413
C7—C8—C9	118.4 (3)	C9—C10—H10	119.203
C8—C9—C10	121.2 (3)	C11—C10—H10	119.200
C9—C10—C11	121.6 (3)	C10—C11—H11	121.583
C10—C11—C12	116.9 (3)	C12—C11—H11	121.565
N1—C12—C7	108.3 (2)	C13—C14—H14	176 (3)
N1—C12—C11	128.6 (3)	C15—C16—H16	121.418
C7—C12—C11	123.1 (3)	C17—C16—H16	121.430
N1—C13—C14	179.6 (3)	C16—C17—H17	119.309
N2—C15—C16	129.0 (3)	C18—C17—H17	119.324
N2—C15—C20	108.4 (3)	C17—C18—H18	119.660
C16—C15—C20	122.6 (3)	C19—C18—H18	119.666
C15—C16—C17	117.2 (3)	C18—C19—H19	120.470
C16—C17—C18	121.4 (3)	C20—C19—H19	120.473
C17—C18—C19	120.7 (3)	C21—C22—H22	120.769
C18—C19—C20	119.1 (3)	C23—C22—H22	120.768
C15—C20—C19	119.1 (3)	C22—C23—H23	119.593
C15—C20—C21	107.3 (3)	C24—C23—H23	119.592
C19—C20—C21	133.5 (3)	C23—C24—H24	119.181
C20—C21—C22	133.4 (3)	C25—C24—H24	119.175
C20—C21—C26	107.3 (2)	C24—C25—H25	121.764
C22—C21—C26	119.3 (3)	C26—C25—H25	121.763
C21—C22—C23	118.5 (3)	C27—C28—H28	176 (3)
			
C1—N1—C12—C7	1.2 (3)	C8—C7—C12—N1	−177.7 (2)
C1—N1—C12—C11	−177.5 (2)	C8—C7—C12—C11	1.1 (4)
C12—N1—C1—C2	178.0 (2)	C12—C7—C8—C9	−0.5 (4)
C12—N1—C1—C6	−0.8 (3)	C7—C8—C9—C10	0.1 (4)
C13—N1—C1—C2	0.9 (4)	C8—C9—C10—C11	−0.3 (4)
C13—N1—C1—C6	−177.9 (3)	C9—C10—C11—C12	0.9 (4)
C13—N1—C12—C7	178.4 (2)	C10—C11—C12—N1	177.3 (3)
C13—N1—C12—C11	−0.3 (4)	C10—C11—C12—C7	−1.3 (4)
C15—N2—C26—C21	0.7 (3)	N2—C15—C16—C17	−180.0 (3)
C15—N2—C26—C25	−179.2 (3)	N2—C15—C20—C19	−179.73 (19)
C26—N2—C15—C16	−179.9 (3)	N2—C15—C20—C21	−0.2 (3)
C26—N2—C15—C20	−0.3 (3)	C16—C15—C20—C19	−0.2 (4)
C27—N2—C15—C16	−1.0 (4)	C16—C15—C20—C21	179.4 (3)
C27—N2—C15—C20	178.6 (3)	C20—C15—C16—C17	0.6 (4)
C27—N2—C26—C21	−178.2 (3)	C15—C16—C17—C18	−0.9 (4)
C27—N2—C26—C25	1.9 (4)	C16—C17—C18—C19	0.8 (4)
N1—C1—C2—C3	−177.9 (3)	C17—C18—C19—C20	−0.4 (4)
N1—C1—C6—C5	178.46 (19)	C18—C19—C20—C15	0.1 (4)
N1—C1—C6—C7	0.0 (3)	C18—C19—C20—C21	−179.4 (3)
C2—C1—C6—C5	−0.4 (4)	C15—C20—C21—C22	−178.8 (3)
C2—C1—C6—C7	−178.8 (3)	C15—C20—C21—C26	0.6 (3)
C6—C1—C2—C3	0.7 (4)	C19—C20—C21—C22	0.7 (5)
C1—C2—C3—C4	−0.3 (4)	C19—C20—C21—C26	−179.9 (3)
C2—C3—C4—C5	−0.5 (4)	C20—C21—C22—C23	−179.6 (3)
C3—C4—C5—C6	0.8 (4)	C20—C21—C26—N2	−0.8 (3)
C4—C5—C6—C1	−0.4 (4)	C20—C21—C26—C25	179.1 (2)
C4—C5—C6—C7	177.5 (3)	C22—C21—C26—N2	178.7 (3)
C1—C6—C7—C8	176.5 (3)	C22—C21—C26—C25	−1.4 (4)
C1—C6—C7—C12	0.7 (3)	C26—C21—C22—C23	1.1 (4)
C5—C6—C7—C8	−1.6 (5)	C21—C22—C23—C24	−0.0 (4)
C5—C6—C7—C12	−177.4 (3)	C22—C23—C24—C25	−0.9 (5)
C6—C7—C8—C9	−175.9 (3)	C23—C24—C25—C26	0.7 (4)
C6—C7—C12—N1	−1.2 (3)	C24—C25—C26—N2	−179.6 (3)
C6—C7—C12—C11	177.6 (2)	C24—C25—C26—C21	0.5 (4)

Symmetry codes: (i) *x*, *y*, *z*−1; (ii) *x*, *y*, *z*+1; (iii) *x*−1/2, −*y*+1/2, *z*−1; (iv) *x*+1/2, −*y*+1/2, *z*+1; (v) *x*−1/2, −*y*+1/2, *z*; (vi) −*x*+1/2, *y*−1/2, *z*−1/2; (vii) −*x*+1/2, *y*−1/2, *z*+1/2; (viii) −*x*+1/2, *y*+1/2, *z*+1/2; (ix) −*x*+1/2, *y*+1/2, *z*−1/2; (x) −*x*+1, −*y*, *z*+1/2; (xi) −*x*+1, −*y*, *z*−1/2; (xii) *x*+1/2, −*y*+1/2, *z*; (xiii) −*x*+1, −*y*+1, *z*−1/2; (xiv) −*x*+1, −*y*+1, *z*+1/2; (xv) −*x*+1/2, *y*−1/2, *z*+3/2; (xvi) −*x*+1/2, *y*−1/2, *z*−3/2; (xvii) −*x*+1/2, *y*+1/2, *z*−3/2; (xviii) −*x*+1/2, *y*+1/2, *z*+3/2.
